# Mosaic RASopathies: comprehensive genetic and clinicopathological analysis for diagnosis and management

**DOI:** 10.3389/fcell.2026.1908228

**Published:** 2026-10-15

**Authors:** Tatiana Belysheva, Tatiana Nasedkina, Ekaterina Zelenova, Vera Semenova, Natalia Semenova, Garik Sagoyan, Irina Kletskaya, Yana Vishnevskaya, Elena Sharapova, Kristina Kulikova, Pavel Vlasov, Alexandra Botkina, Margarita Belogurova, Marina Dorofeeva, Timur Valiev, Svetlana Varfolomeeva

**Affiliations:** 1 N.N. Blokhin National Medical Research Center of Oncology, Moscow, Russia; 2 Central State Medical Academy, Moscow, Russia; 3 Engelhardt Institute of Molecular Biology of the Russian Academy of Sciences, Moscow, Russia; 4 Dmitry Rogachev National Medical Research Center of Pediatric Hematology, Oncology and Immunology, Moscow, Russia; 5 Research Center for Medical Genetics, Moscow, Russia; 6 Russian Children’s Clinical Hospital of Pirogov Russian National Research Medical University, Moscow, Russia; 7 Endocrinology Research Centre, Moscow, Russia; 8 N. N. Burdenko National Medical Research Center of Neurosurgery, Moscow, Russia; 9 St.Petersburg Clinical Scientific and Practical Centre for Special Types of Medical Care (Oncology) Named After N.P.Napalkov, St. Petersburg, Russia; 10 Sechenov First Moscow State Medical University, Moscow, Russia

**Keywords:** children, epidermal nevus syndromes, genotype-phenotype correlation, mosaic RASopathy, phacomatosis pigmentokeratotica, rhabdomyosarcoma, Schimmelpenning–Feuerstein–Mims syndrome

## Abstract

**Introduction:**

Mosaic RASopathies are rare conditions caused by postzygotic mutations arising during embryonic development in genes of the RAS/MAPK signaling pathway. They are mainly characterized by a combination of epidermal nevi and extracutaneous manifestations. Distinguishing between different entities can be challenging because of their substantial clinical and genetic overlap. Our aim was to demonstrate the importance of genetic background for accurate diagnosis and further prognostic assessment of disease progression by analyzing genotype–phenotype correlations within this clinical group.

**Methods:**

The study enrolled 18 patients (10 male and 8 female patients) ranging in age from 1 month to 21 years with congenital craniofacial nevi and variable extracutaneous abnormalities. DNA isolated from peripheral blood leukocytes and affected tissues (nevus and tumor) was analyzed using a targeted next-generation sequencing (NGS) panel covering RAS/MAPK pathway genes, followed by Sanger sequencing.

**Results:**

Eight patients were diagnosed with Schimmelpenning–Feuerstein–Mims syndrome caused by postzygotic pathogenic/likely pathogenic variants in the *HRAS* (n = 4) or *KRAS* (n = 4) genes. Seven patients were clinically diagnosed with phacomatosis pigmentokeratotica (PPK), and mosaic genetic variants were identified in the *HRAS* (n = 4), *KRAS* (n = 2), or *BRAF* (n = 1) genes. In two additional patients with similar phenotypes, somatic mutations in *PIK3CA* (n = 1) and *FGFR2* (n = 1) were identified in affected skin, while in one further case, no clinically significant variant was detected.

**Discussion:**

We compared the spectrum of extracutaneous anomalies according to the affected gene by combining our data with data from other clinical cases of mosaic RASopathies described in the literature. In summary, 53 cases with *HRAS* postzygotic variants and 41 cases with *KRAS* variants were included in the analysis of genotype–phenotype correlations. Hypophosphatemic rickets (HR) and malignant tumors were associated with pathogenic/likely pathogenic variants in the *HRAS* gene, while ophthalmological, neurological, cardiovascular, and renal disorders were more common in *KRAS*-mutant patients.

**Conclusion:**

Thus, genetic testing of affected tissue in comparison to normal tissue (peripheral blood) is essential for accurate diagnosis of these mosaic disorders. Consideration of clinical manifestations and the identified genotype–phenotype correlations may further assist in determining prognosis and guiding patient management.

## 1 Introduction

Molecular genetic studies of epidermal nevus syndromes (ENSs) clearly defined them as mosaic disorders caused by postzygotic pathogenic/likely pathogenic variants in various genes, primarily those involved in the RAS/MAPK signaling pathway (Waldman et al., 2022; Groesser et al., 2012; Jaeger et al., 2025). Epidermal nevi (EN) are cutaneous hamartomas that include keratinocytic or organoid nevi and may involve melanocytes or other skin appendages (epidermis, hair follicles, and sweat and sebaceous glands). Beyond skin lesions that present as various types of nevi, these syndromes are characterized by a wide range of extracutaneous abnormalities affecting virtually all organ systems. Currently, however, the nevus subtype (sebaceous, spilus, melanocytic, *etc.*) remains the main diagnostic feature used to classify the disease (Nicholson and Daveluy, 2025).

At the same time, this group of mosaic disorders shows substantial genetic and clinical overlap. Congenital craniofacial linear nevus sebaceous (LNS) is a hallmark of Schimmelpenning–Feuerstein–Mims syndrome (SFMS), but it is also a necessary component of phacomatosis pigmentokeratotica (PPK) when it occurs in association with a nevus spilus (speckled lentiginous nevus) (Groesser et al., 2013; Torchia and Happle, 2019). The combination of EN and hypophosphatemic rickets (HR) is classified as cutaneous skeletal hypophosphatemia syndrome (CSHS) (Lim et al., 2014). Genetic overlap between these clinical subtypes has been identified, and the same mosaic pathogenic/likely pathogenic variants in RAS/MAPK pathway genes, primarily *HRAS* and *KRAS*, have been found in SFMS, PPK, CSHS, keratinocytic epidermal nevus (KEN), congenital melanocytic nevus, and other conditions (Groesser et al., 2012; Groesser et al., 2013; Hafner and Groesser, 2013; Igawa et al., 2016; Boppudi et al., 2016; Kuroda et al., 2015; Sorlin et al., 2021). The same *KRAS* variants are present in oculoectodermal syndrome (OES) and encephalocraniocutaneous lipomatosis (ECCL); the latter is also characterized by nevus sebaceous, typically in obligatory combination with nevus psiloliparus; congenital aplasia cutis, alopecia, and skin pigmentation are also common additional features. Pathogenic/likely pathogenic variants in other genes, such as *BRAF*, *PIK3CA*, *FGFR1-3*, and *PTEN*, may also be detected in nevi and can be accompanied by syndromic manifestations (Jaeger et al., 2025).

Recent studies have suggested that the genes involved play a key role in determining the nature of extracutaneous disorders, disease prognosis, and possible treatment options (Zelenova et al., 2025a; Wu et al., 2025). There is strong evidence for the significance of genetic classification, which encompasses ENS under the general term “mosaic RASopathies” (Hafner and Groesser, 2013; Morren et al., 2024; Maridet et al., 2018; Chang et al., 2021; Davies et al., 2022). To date, the fundamental mechanisms underlying the development of ENS, along with the reasons for the variability of clinical manifestations, remain largely unknown. We previously conducted a systematic review of genetically confirmed clinical cases of SFMS and identified correlations between the presence of *KRAS* or *HRAS* pathogenic variants and specific phenotypic manifestations (Zelenova et al., 2025a).

In this study, we present a series of 18 clinical cases with cutaneous manifestations and overlapping phenotypes characteristic of mosaic RASopathies to demonstrate the importance of genetic testing for accurate diagnosis and predicting the extracutaneous phenotypic manifestations most typically associated with pathogenic variants in various genes of the RAS/MAPK signaling pathway. We also analyzed an expanded cohort of patients diagnosed with SFMS and PPK, using data from previously described clinical cases with a genetically confirmed diagnosis, in order to more clearly delineate the genotype–phenotype relationship. These data provide a better understanding of the molecular basis of mosaic diseases and may also have implications for patient management. An earlier version of this work has been previously deposited as a preprint (Belysheva et al., 2026).

## 2 Materials and methods

### 2.1 Study population

The study included 18 patients (10 male and 8 female patients) ranging in age from 1 month to 21 years with clinical features of ENS. Next-generation sequencing (NGS) was performed on DNA isolated from blood leukocytes, nevus tissue, normal skin, and tumor tissue using the QIAamp DNA Mini Kit (QIAGEN, Hilden, Germany). Genetic testing of blood (normal tissue) was performed for all patients in order to exclude germline variants associated with hereditary syndromes that have similar phenotypes.

### 2.2 Next-generation sequencing

Libraries were prepared using the KAPA HyperPrep Kit (Roche, Basel, Switzerland) according to the manufacturer’s protocol and hybridized to customized capture probes targeting the coding regions of genes of the RAS/MAPK signaling pathway. For a subset of patients, clinical exome sequencing of blood samples was carried out using the TruSight One Expanded Sequencing Panel (Illumina, San Diego, CA, United States), with libraries prepared using the Illumina DNA Prep with Enrichment kit (Illumina, San Diego, CA, United States) per the manufacturer’s instructions. Sequencing was performed on a NextSeq 2000 instrument (Illumina, San Diego, CA, United States) using the P1 300 cycle kit (Illumina, San Diego, CA, United States). The average coverage was 250–350×, with ≥90% of the target region covered at least 50× and a quality score of ≥99.9%. The identified pathogenic/likely pathogenic variants were verified by Sanger sequencing.

Sequencing data were processed using an automated pipeline that included read alignment to the reference human genome (hg38), post-alignment processing, variant detection, and quality filtering. Single-nucleotide variants and small indels were identified using GATK with the GRCh38/hg38 reference. To ensure analytical sensitivity, we applied a minimum variant allele frequency (VAF) threshold of ≥2% for initial variant calling, chosen based on the average sequencing depth (>200×) and base quality metrics (Phred score ≥30). The annotation and interpretation of the detected variants were performed as described previously (Semenova et al., 2026). All candidate variants were validated by Sanger sequencing using a 3500 Series Genetic Analyzer (Applied Biosystems, Thermo Fisher Scientific, Waltham, MA, United States).

### 2.3 Statistical analysis

Statistical analysis was performed using Python version 3.12 (Python Software Foundation, Wilmington, DE, United States) with the following open-source libraries: pandas (version 2.2.0) for data manipulation, SciPy (version 1.13.0) for Fisher’s exact test, and statsmodels (version 0.14.1) for multiple-testing correction. Fisher’s exact test (two-sided) was used to compare the prevalence of each extracutaneous disorder between *HRAS*- and *KRAS*-mutant patients. The odds ratio (OR) and 95% confidence interval (CI) were calculated with *KRAS* as the reference group (*KRAS*/*HRAS*), such that an OR > 1 indicates a higher prevalence in *KRAS*-mutant patients. For contingency tables containing zero cells (i.e., rickets), the Haldane correction (+0.5) was applied to all cells to enable stable estimation of ORs and CIs. The standard error of the log odds ratio was calculated using the Woolf method.

To account for multiple comparisons across the 11 clinical features examined, raw *p*-values were adjusted using the Benjamini–Hochberg procedure to control the false discovery rate (FDR) at a threshold of q < 0.05. Differences with FDR-adjusted *p*-values of q < 0.05 were considered statistically significant. All analyses were performed in a reproducible Jupyter Notebook environment, and the code is available upon request.

### 2.4 Review of the literature

A systematic literature review was conducted using PubMed and ResearchGate for the period 1946–2025. The following search terms were used: “epidermal nevus syndrome,” “Schimmelpenning–Feuerstein–Mims,” “phacomatosis pigmentokeratotica,” “mosaic RAS pathology,” “mosaic phacomatosis,” and “postzygotic/mosaic mutation in *HRAS/KRAS/NRAS*.” Full-text articles describing clinical case reports, case series, or cohort studies were included. The eligibility criteria were as follows: each article had to provide information on patient age and sex, the distribution of affected skin areas, the presence of extracutaneous anomalies (including malignant tumors), and the results of genetic testing (tissue type analyzed and genetic variant identified). Exclusion criteria were incorrect diagnosis and a lack of clinical or genetic data. We also excluded articles describing patients with OES and ECCL as these are distinct entities with characteristic phenotypes within the ENS group. Duplicate cases were excluded. The phenotypic characteristics of patients reported in the literature were extracted from the clinical descriptions of each case and by reviewing photographic materials. If an organ system was not described in an article, it was assumed that no pathological changes were present in that organ system.

## 3 Results

In total, 18 patients were included in the study. The diagnosis was assigned based on the nevus type, extracutaneous abnormalities, and genetic data. The clinical and genetic characteristics of the patients are presented in Supplementary Table S1 (Supplementary Material). A more detailed description of these clinical cases is provided below.

### 3.1 Schimmelpenning–Feuerstein–Mims syndrome

Patient ID1 was a 9-year-old boy with congenital craniofacial LNS and EN on the trunk (Figures 1A–D). Subsequently, numerous Spitz nevi developed on his face, some of which were surgically excised (Figures 1E–G). The patient had delayed physical development, and from 12 months of age, he developed skeletal deformities (Figures 1C,D); radiographs showed dysplastic foci of mixed lytic and sclerotic bone and classic signs of rickets. Treatment for HR (phosphate and alfacalcidol) was ineffective, so at 4 years of age, targeted therapy with burosumab (a monoclonal antibody against fibroblast growth factor 23) was started (Kulikova et al., 2023). After 15 months of treatment, pain was relieved, independent walking was restored, and biochemical values (alkaline phosphatase [ALP], serum phosphorus, and calcium) normalized. Exome sequencing of blood DNA did not reveal any clinically significant genetic variant. Genetic testing of the nevus identified a pathogenic *HRAS* variant, c.182A>G (p.Gln61Arg), with a variant allele frequency (VAF) of 62%.

**FIGURE 1:**
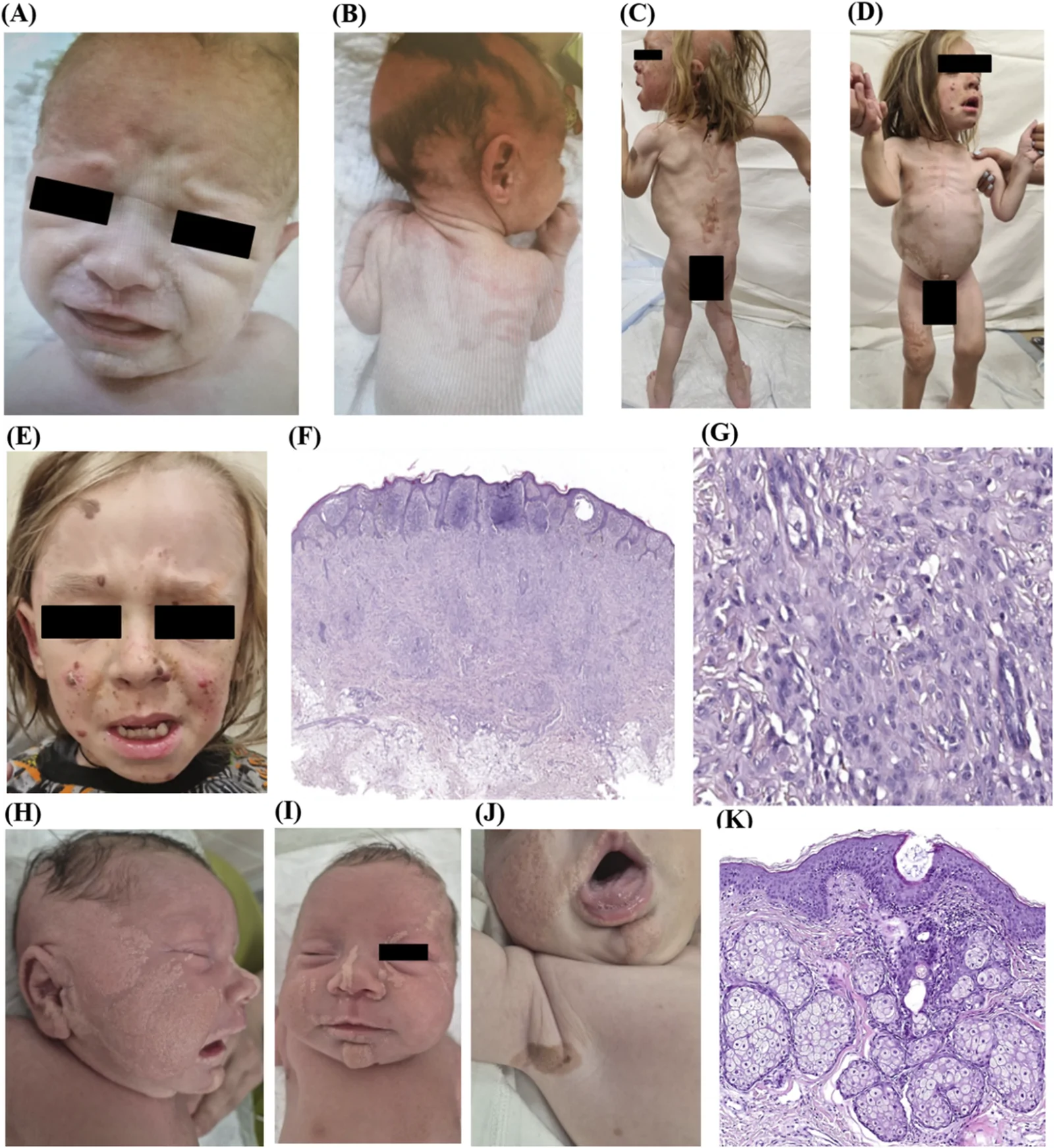
Phenotype ID1 **(A–E)**. **(A and B)** LNS on the head, trunk, and limbs, predominantly right-sided, following Blaschko’s lines at 2 months; **(C and D)** multiple deformities of the long bones and chest at 1 year; **(E)** Spitz nevi on the face and small congenital melanocytic nevi on the forehead at 1 year; histology ID1 **(F and G)**. **(F)** Spitz nevus: a symmetrical, well-demarcated, wedge-shaped lesion with marked acanthosis (H&E, ×150); **(G)** the lesion is composed of epithelioid cells with abundant cytoplasm and monomorphic nuclei, and the stroma is slightly fibrotic in the reticular dermis (H&E, ×350). Phenotype ID2 **(H–J)**. **(H and I)** LNS on the head and face, predominantly right-sided, following Blaschko’s lines, and right ptosis; **(J)** linear pigmented nevus in the axillary region; histology ID2: **(K)** nevus sebaceous of Jadassohn with papillomatosis and hyperkeratosis, hamartomatous sebaceous glands located higher than normal in the dermis and opening directly onto the epidermal surface (H&E, ×250).

Patient ID2, an 8-month-old girl, had craniofacial LNS (Figures 1H–K), a pigmented nevus in the right axillary region (Figure 1J), right palpebral ptosis, and a patent ductus arteriosus present since birth. MRI showed moderate enlargement of the subarachnoid spaces. At 3 months of age, the patient was diagnosed with paroxysmal atrial tachycardia with atrial flutter and aplasia cutis on the vertex. Genetic testing of the LNS revealed the same *HRAS* variant, c.182A>G (p.Gln61Arg) (VAF 28%–36%). The patient is being followed up due to the possible risk of future complications.

Patient ID3, a 4-year-old boy, had LNS on the head, face, trunk, and extremities (Figures 2A–D). He spent the first 3 months in the intensive care unit due to central nervous system (CNS) depression, pseudobulbar syndrome, and aspiration pneumonia. MRI revealed encephalopathy, and at 4 months of age, seizures were recorded. The patient had also aortic stenosis, left ventricular hypertrophy, and a patent foramen ovale. At 6 months of age, he underwent left orchiopexy; during surgery, a soft tissue tumor in the paratesticular region was found and subsequently removed. The tumor was identified as alveolar rhabdomyosarcoma (RMS), T1N1M1, IRS group IV, without *FOXO1* gene rearrangements (a favorable prognostic sign) and with metastases to the soft tissues of the left femur and right hand (Figures 2E–G). After nine courses of adjuvant polychemotherapy (PCT), the patient has remained in remission for over 2 years. At 20 months of age, central precocious puberty (CPP) was diagnosed, and triptorelin acetate was prescribed. He also sustained a fracture of the left femur; elevated ALP, hypophosphatemia, and phosphaturia were detected, indicating HR. Phosphate and alfacalcidol were prescribed, and burosumab was started at 3 years and 9 months of age. After 6 months of treatment, laboratory values normalized, but there was no clinical improvement. A pathogenic variant in *HRAS* c.37G>C (p.Gly13Arg) was detected, with a VAF ranging from 17% to 22% in the nevus and 84% in the tumor.

**FIGURE 2:**
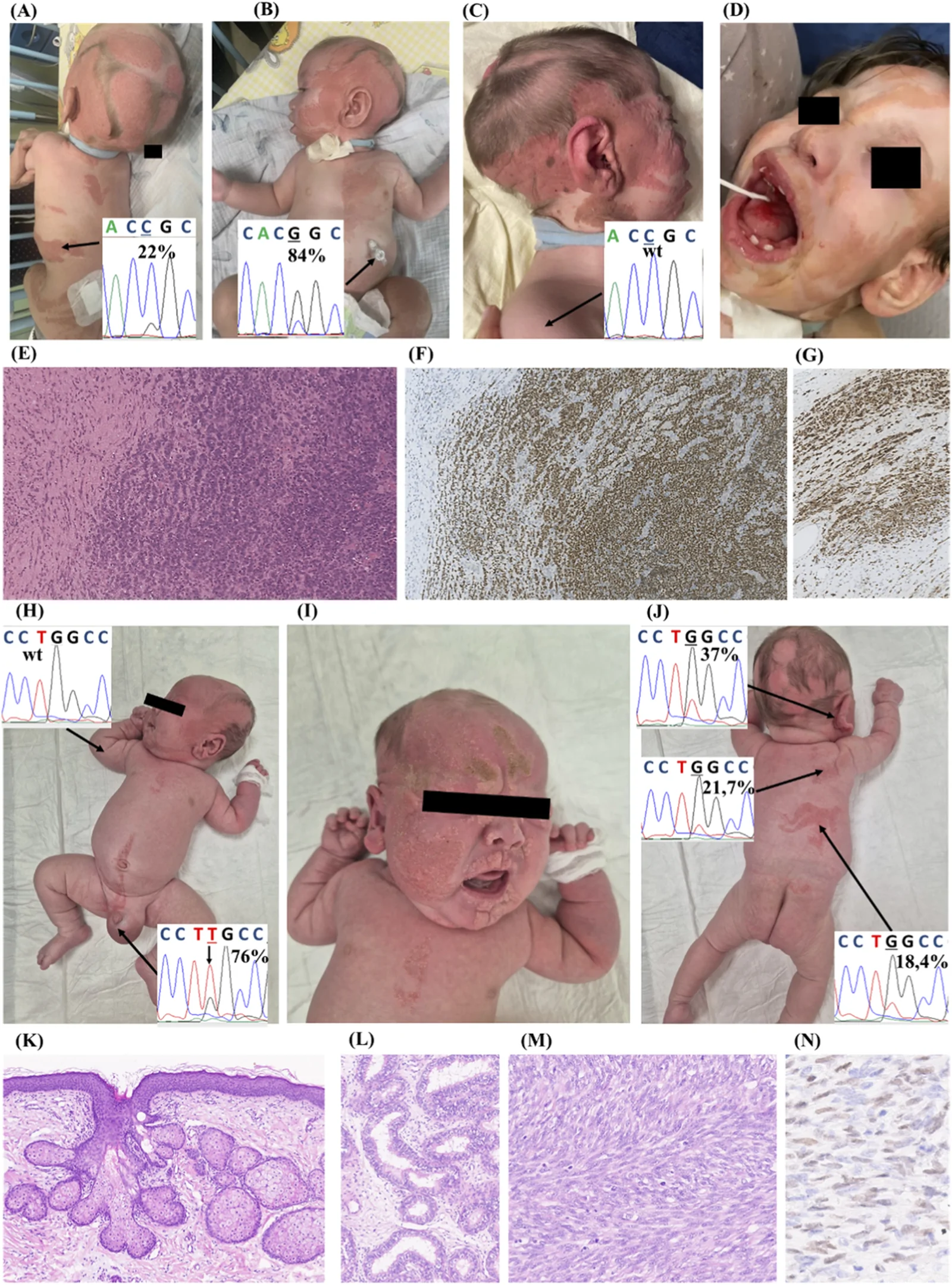
Phenotype ID3: **(A–D)** LNS on the scalp, face, neck, and trunk, predominantly left-sided, following Blaschko’s lines; a mosaic variant in the *HRAS* gene with a VAF of 22% was detected in the nevus tissue **(A)** and a VAF of 84% in the tumor **(B)** but not in the biopsy of healthy skin **(C)**. **(D)** Severe oral papillomatosis. Histology ID3: **(E)** trabecular-solid round-cell tumor (H&E, ×100). Immunohistochemistry ID3 **(F–G)**. **(F)** Diffuse expression of MyoD1 (×100) in the tumor; **(G)** diffuse expression of desmin (×200) in the tumor. Phenotype ID4: **(H–J)** LNS on the face, scalp, and trunk, predominantly right-sided and in the midline, following Blaschko’s lines; Sanger sequencing results indicating the VAF at the loci studied. Histology ID4: **(K–M)**. **(K)** Hyperplastic sebaceous glands without excretory ducts and with underdeveloped hair follicles are observed in the skin (H&E, ×100); **(L)** moderate testicular hypoplasia, with seminiferous tubules unevenly spaced due to stromal sclerosis, disruption of layer stratification in the germinal epithelium, cells with light cytoplasm, and cells with dystrophic changes; **(M)** testicular RMS, spindle cell variant (H&E, ×200). Immunohistochemistry ID4: **(N)** focal expression of MyoD1 (×200) in tumor cells.

Patient ID4, a 1-year-old boy, had congenital LNS on the scalp, face, and trunk (Figures 2H–N). Other findings included a patent foramen ovale, cardiomegaly, right cryptorchidism, and an enlarged left testis. At 1 month of age, a highly vascularized scrotal tumor was detected (spindle cell RMS, T1N0M0, IRS group IA); left orchiectomy was performed, followed by PCT. At 4.5 months of age, epilepsy was diagnosed, and anticonvulsant treatment was started. A pathogenic *HRAS* variant, c.181C>A (p.Gln61Lys), was identified, with a VAF ranging from 18% to 37% in the nevus and 76% in the tumor.

Patient ID5, a 14-year-old girl, had at birth LNS on the scalp and face, ptosis, deformity of the right palpebral region (Figures 3A–D), and a congenital heart defect. At 1 month of age, clipping of the ductus arteriosus was performed, and an orbital lipoma was removed; over the next 2 years, multiple papillomatous lesions of the skin and palate were excised. At 2 years of age, because of suspected Wilms’ tumor, a right nephrectomy was performed, but histology confirmed nephroblastomatosis. At 2.5 years of age, gliosis in the right occipitoparietal region and an arachnoid cyst were detected on MRI; focal epilepsy was diagnosed but responded poorly to treatment. At 3 years of age, the patient had a stroke with right-sided hemiparesis and another cerebrovascular accident 6 months later. Hyperhomocysteinemia (homocysteine 12 μmol/L) was detected and treated with dipyridamole. At the same age, CPP (thelarche and adrenarche) was observed; several benign tumors appeared, including an extramedullary neoplasm of the C2–C3 vertebrae, syringocystadenoma papilliferum within the LNS, and a plexiform neurofibroma, which was excised at 7 years of age (Figures 3E–G). Currently, sexual development has normalized, but the patient has severe neurological and intellectual impairments. Exome sequencing of blood DNA did not reveal any pathogenic variants. A pathogenic variant, c.35G>A (p.Gly12Asp), in the *KRAS* gene was identified in the nevus (VAF 35%).

**FIGURE 3:**
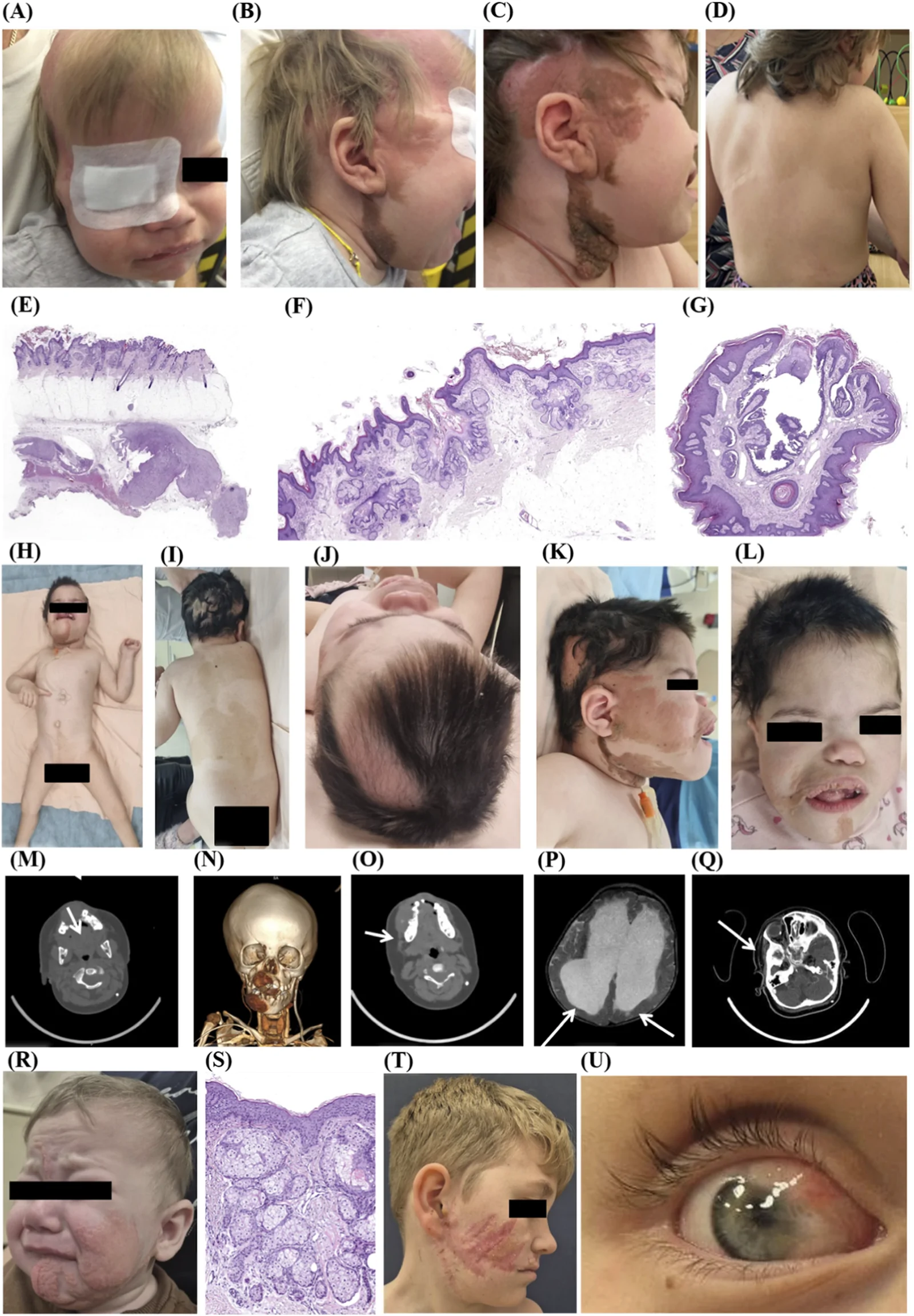
SFMS patients with a *KRAS* variant. Phenotype ID5 at age 2 **(A and B)** and 4 years **(C and D)**. **(A–C)** LNS on the scalp, face, and neck, predominantly right-sided, following Blaschko’s lines; **(D)** a single café-au-lait macule on the right shoulder and scapula. Histology **(E–G)**. **(E)** Nevus sebaceous of Jadassohn with underlying plexiform neurofibroma (H&E, ×150); **(F)** marked papillomatosis and hyperkeratosis, with hamartomatous sebaceous glands located higher than normal in the dermis, without connection to a hair follicle and with direct opening onto the epidermal surface (H&E, ×250); **(G)** syringocystadenoma papilliferum presenting as focal cystic invaginations of the infundibular epithelium projecting into the dermis, covered by a double layer of columnar and cuboidal cells (H&E, ×350). Phenotype ID6 (H–L). **(H and I)** Café-au-lait macules on the trunk; **(J–L)** LNS on the scalp, face, neck, and trunk, predominantly right-sided, following Blaschko’s lines. Brain MRI ID6 **(M–Q**). **(M–O)** Granulosa cell tumor (indicated by arrows); **(P)** white matter atrophy (indicated by arrows); **(Q)** retinal calcifications OU (oculus uterque) (indicated by arrows). Phenotype ID7: **(R)** LNS on the face following Blaschko’s lines. Histology ID7: **(S)** hamartomatous sebaceous glands opening directly onto the epidermal surface (H&E, ×250). Phenotype ID8 **(T, U)**. **(T)** LNS on the right side of the face after an attempt to surgically excise the nevus at a local hospital; **(U)** partially vascularized corneal opacity in the right eye after removal of the epibulbar dermoid.

Patient ID6, a 5-year-old girl, was born at 36 weeks of gestation with low birth weight (1880 g) and length (42 cm). At birth, LNS was present on the scalp, face, and neck (Figures 3H–Q). At 4 months of age, she developed seizures; MRI revealed bilateral lipomatous lesions of the trigeminal nerves, ventriculomegaly, dysplasia of the right temporo-parieto-occipital region, white matter atrophy, and hydrocephalus. Focal epilepsy was subsequently diagnosed, and antiepileptic therapy had little effect. At 6 months of age, echocardiography revealed a rhabdomyoma in the right ventricle; a central giant cell granuloma of the right upper and lower jaws was also diagnosed, but surgery was postponed until her somatic status improved. The patient has severe cognitive impairments. Ocular abnormalities included epibulbar/corneal dermoids, bilateral retinal calcifications, and strabismus. A pathogenic variant in *KRAS* c.35G>A (p.Gly12Asp) was detected in the nevus (VAF 23%–28%).

Patient ID7, a 6-month-old boy, had congenital LNS confined to the face (Figures 3R,S). Genetic testing of the nevus revealed a pathogenic variant in *KRAS* c.35G>A (p.Gly12Asp), which was not detected in other tissues. He is currently under regular follow-up for suspected SFMS.

Patient ID8, a 12-year-old boy, had congenital LNS on the right side of the face (Figure 3T). At a local hospital, an attempt was made to partially remove the nevus surgically. He had had epibulbar dermoids in the right eye since birth, and these were removed at 5 months. He also had mixed intermittent monocular non-accommodative strabismus and partial, vascularized corneal opacity in the right eye (Figure 3U). At 1.5 months of age, he was diagnosed with epilepsy, and at 4 years, resection of the temporal lobe was performed, followed by histological examination, which revealed a gangliocytoma (WHO grade 1). Since then, he has not experienced seizures; however, significant developmental delay was observed (he started walking at 1 year and 7 months and speaking at 5 years). Genetic testing of nevus tissue revealed a pathogenic *KRAS* variant, c.35G>A (p.Gly12Asp), which was not observed in blood DNA or unaffected skin.

### 3.2 Phacomatosis pigmentokeratotica

Patient ID9, a 9-year-old girl, was born with LNS involving the scalp and face and a nevus spilus on the trunk and limbs (Figures 4A–C). At 4 months of age, she had seizures and was prescribed tetracosactide with a positive effect. At 8 months, a hypervascular tumor (presumed to be RMS after biopsy) with invasion of the bladder and vagina was identified. She received nine courses of neoadjuvant PCT, after which cystectomy, hysterectomy, and vaginal resection were performed, and the tumor was diagnosed as pelvic ectomesenchymoma, T2bN1M0, IRS-IIIA (Figures 4D–K). A patent foramen ovale was also noted. A pathogenic *HRAS* variant, c.37G>C (p.Gly13Arg), was detected (VAF 25%–36% in the nevus and 41% in the tumor).

**FIGURE 4:**
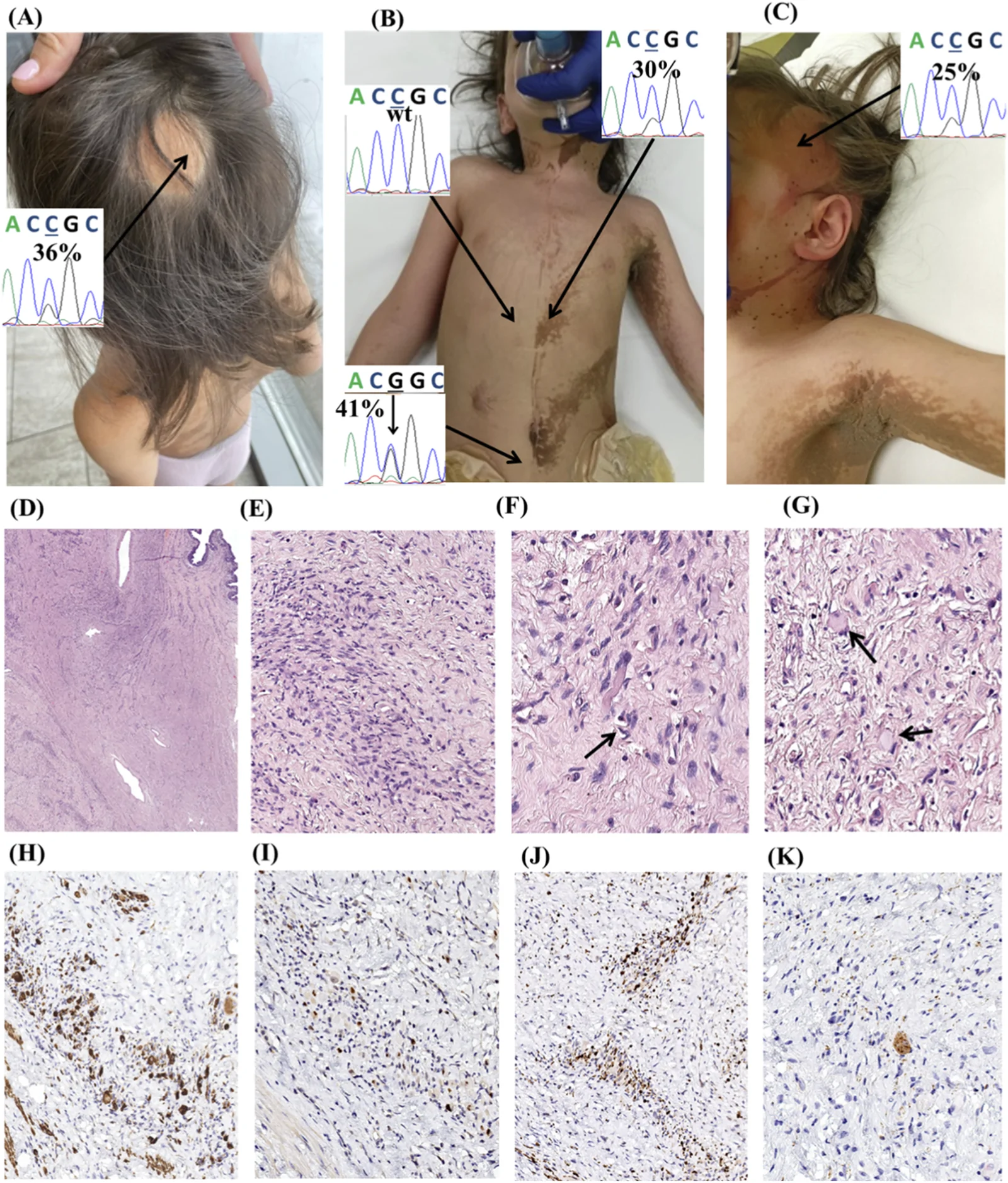
Phenotype ID9: **(A–C)** LNS on the scalp, face, and neck, as well as on the chest and abdomen, predominantly on the left side and in the left axillary region, following Blaschko’s lines; nevus spilus on the left side of the neck. Histology ID9 **(D–G)**. **(D)** Ectomesenchymoma in the urinary bladder wall (H&E, ×80); **(E)** neoplastic tissue shows moderate-to-low cellularity and a biphasic structure (H&E, ×250); **(F)** the rhabdomyoblastic component is composed of round to oval cells with abundant eosinophilic cytoplasm and cross striations (arrowhead) (H&E, ×350); **(G)** the second component is composed of spindle cells with wavy nuclei and admixed ganglion cells (arrowhead) (H&E, ×350). Immunohistochemistry ID9 **(H–K)**. **(H)** expression of desmin (×350); **(I)** expression of myogenin in the rhabdomyoblastic component (×350); **(J)** expression of S100 in the ganglioneuroma-like component (×350); **(K)** expression of synaptophysin in ganglion cells in the ganglioneuroma-like component (arrowhead) (×350).

Patient ID10, a 19-year-old boy, had extensive congenital LNS and later developed widespread nevus spilus and café-au-lait macules on the body (Figures 5A–F). At 8 years of age, he underwent surgery with a transpedicular system due to severe scoliosis (Gamayunov et al., 2016). He also had corneal opacity in the left eye, delayed speech development, and intellectual disability. A pathogenic *HRAS* variant, c.37G>C (p.Gly13Arg), was identified in the nevus (VAF 37%).

**FIGURE 5:**
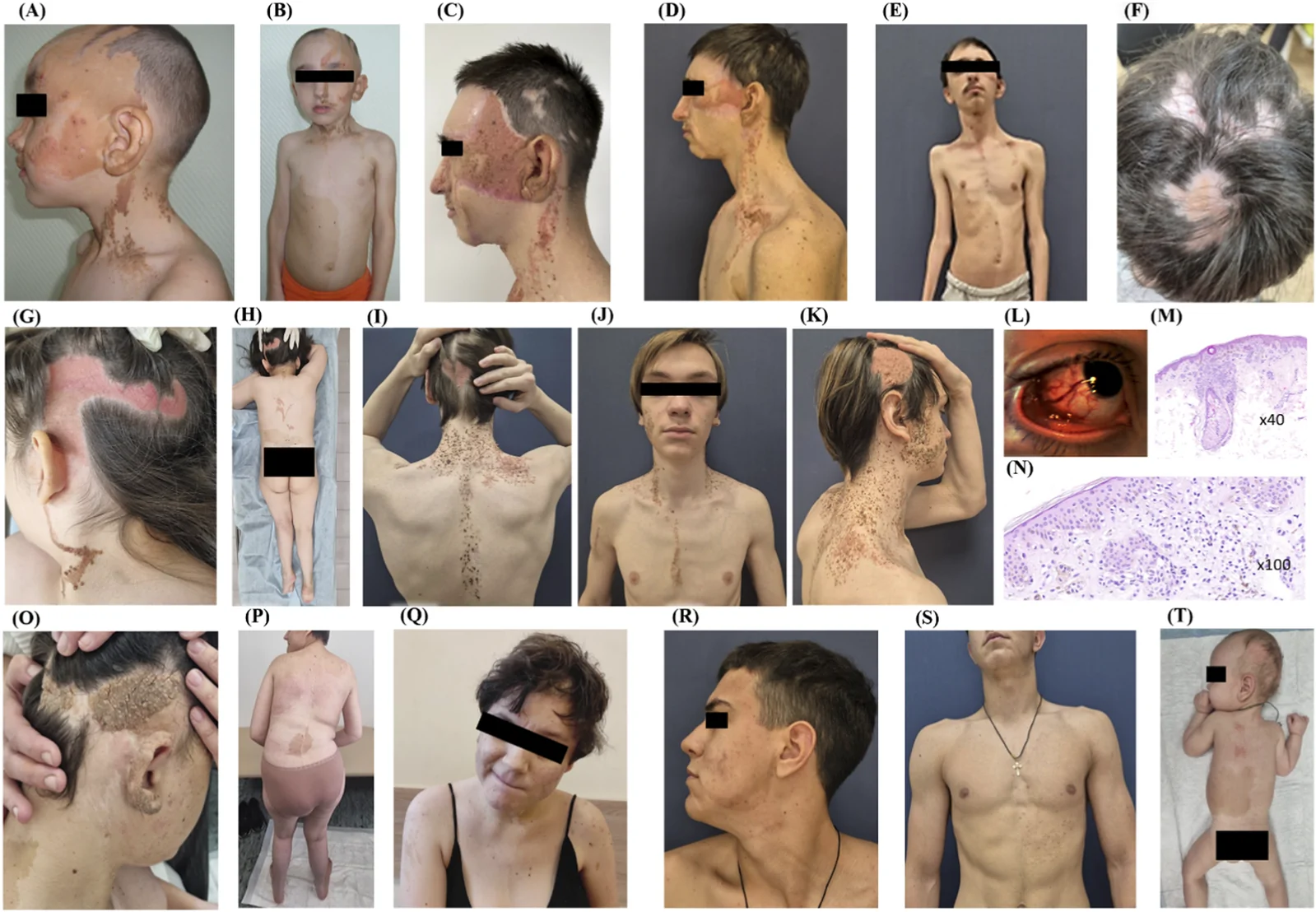
Phenotype ID10 at ages 3 **(A and B)** and 18 **(C–F)** years. LNS on the scalp, face, neck, and trunk, predominantly left-sided, following Blaschko’s lines, and nevus spilus on the right side of the trunk and left arm, with pronounced scoliotic deformities. Phenotype ID11 **(G and H). (G)** LNS on the scalp, neck, auricle, and trunk, predominantly left-sided, following Blaschko’s lines; **(H)** pronounced scoliosis of the spine and nevus spilus in the lumbar region. Phenotype ID12 **(I–N)**. LNS on the scalp and nevus spilus on the face, neck, back, and chest **(I–K)** and hemangioma of the right eye **(L)**. Nevus spilus, histology **(M, N)** against a background of slight epidermal melanocytic hyperplasia: there are multiple compound melanocytic nevi (**M**, H&E, ×40), composed of junctional and dermal nests of small monomorphic nevus cells with periadnexal distribution (**N**, H&E, ×100). Phenotype ID13 **(O–Q)**. LNS observed on the scalp, neck, and back; nevus spilus on the face, left arm, and back. Phenotype ID14 **(R, S)**. LNS on the scalp, face, and neck, predominantly left-sided, following Blaschko’s lines; nevus spilus on the left side of the chest. Phenotype ID15 **(T)**. Facial dysmorphism (high forehead and hypoplasia of the brow ridges, depressed bridge of the nose), nevus spilus on the trunk, and LNS on the scalp and trunk in the midline.

Patient ID11, a 14-year-old girl, had LNS since birth, and later a nevus spilus developed in the lumbar region (Figures 5G,H). At 2 years of age, the first fracture occurred, and HR was diagnosed. The patient received phosphate and alfacalcidol for 8 years but had multiple fractures and shortening of the left leg by 8 cm. From the age of 11 years, her mobility became limited, and she currently receives burosumab. A pathogenic *HRAS* variant, c.182A>G (p.Gln61Arg), was identified in the nevus tissue (VAF 33%). She is currently under follow-up without other abnormalities.

Patient ID12, a 17-year-old boy, had LNS on the scalp and a nevus spilus on the right side of the face, as well as on the midline of the neck, back, and chest since birth (Figures 5I–N). Ophthalmologic examination revealed a hemangioma of the right eye. Brain MRI showed cerebellar ectopia into the foramen magnum and vascular anomalies (hypoplasia of the proximal part of the left anterior cerebral artery and anterior trifurcation of the right internal carotid artery). At the age of 14 years, he was also diagnosed with a bone cyst in the upper part of the right humerus. No pathological changes in other organ systems were observed. Genetic examination of both types of nevi revealed a pathogenic *HRAS* variant, c.182A>G (p.Gln61Arg), which was not observed in other tissues or blood.

Patient ID13, a 21-year-old girl, was born with LNS on the scalp, ear, and neck (Figures 5O–Q). Later, facial angiofibromas appeared, and a nevus spilus was detected on the face and trunk. From 2 months of age, she began to suffer from pharmacoresistant epilepsy. MRI revealed focal cortical dysplasia in the right frontotemporal lobe, and at 5 years of age, a lobectomy was performed. At 14 years, she underwent embolization of an arteriovenous malformation at the level of the T4–T8 vertebrae, and 3 years later, angiomyolipomas and a renal cyst were identified. Retinal hamartomas and mild pulmonary valve stenosis were also found. The patient has poor social functioning and receives antipsychotic therapy (risperidone) due to self-aggression. Exome sequencing of blood DNA did not reveal any pathogenic variants, but targeted sequencing of the nevus identified a pathogenic *KRAS* variant, c.35G>A (p.Gly12Asp), with a VAF of 32%.

Patient ID14, an 18-year-old boy, had LNS on the face and back and a nevus spilus on the chest at birth (Figures 5R,S). At the age of 1 year, his left lower extremity became swollen, and at 4 years, a venous malformation was detected by MR angiography and surgically corrected. Brain MRI revealed hypoplasia of the left anterior cerebral artery and aplasia of the left posterior communicating artery. No other abnormalities were identified. A pathogenic *KRAS* variant, c.34G>A (p.Gly12Ser), was identified in the nevus (VAF 30%). Because clinical features of both Parkes Weber syndrome (venous malformation of the leg and hypoplasia of the cerebral arteries) and PPK (congenital LNS and nevus spilus) were present simultaneously, he was diagnosed with a combination of the two syndromes.

Patient ID15, a 1-year-old boy, was born with congenital LNS on the scalp and neck (Figure 5T). At the age of 1 month, a nevus spilus appeared in the lumbar region. Subsequently, the patient was diagnosed with focal epilepsy, hypoplasia of the corpus callosum, a retrocerebellar cyst, nystagmus, strabismus, and sensorineural hearing loss. He also suffered from severe recurrent infections and died from pneumonia at the age of 2 years. Primary immunodeficiency or other hereditary diseases were excluded by exome sequencing of blood DNA. A likely pathogenic *BRAF* variant, c.1406G>C (p.Gly469Ala, rs121913355), was found only in the nevus (VAF 26%). The differential diagnosis between the mosaic form of cardiofaciocutaneous (CFC) syndrome and PPK in this patient was discussed (Zelenova EE. et al., 2025).

### 3.3 Other clinical cases

Patient ID16, a 10-year-old girl (Figures 6A,B), had an EN on the scalp, face, and neck since birth (Ol’shanskaya et al., 2017). At 2 months of age, focal epilepsy was diagnosed, and MRI revealed hemimegalencephaly. At 10 years of age, she was found to have left-sided hemiparesis, delayed psychomotor development, strabismus of the left eye, and nystagmus. A pathogenic *PIK3CA* variant, c.1633G>A (p.Glu545Lys), was identified in the nevus (VAF 29%), indicating a diagnosis of *PIK3CA*-Related Overgrowth Spectrum (PROS).

**FIGURE 6:**
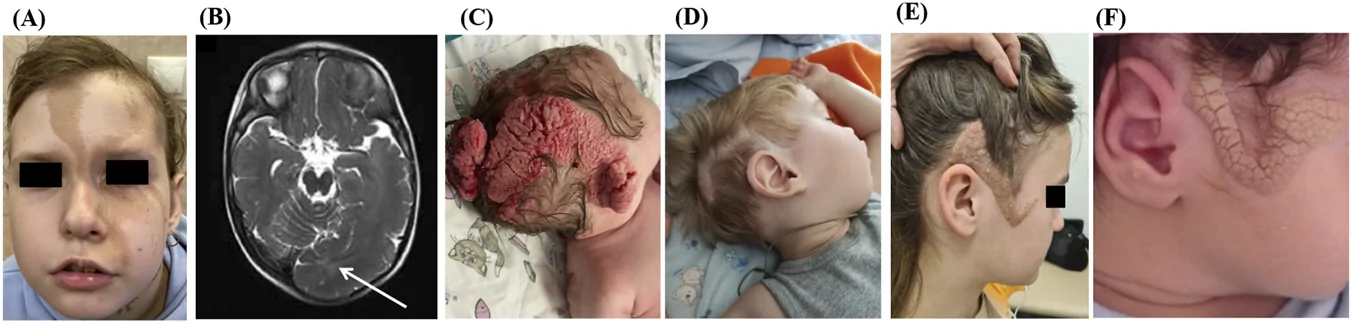
Differential diagnosis. Phenotype ID16 **(A)**. Epidermal nevus on the face and neck, more pronounced on the left side. Brain MRI ID16 **(B)**. Left-sided hemimegalencephaly (enlarged hemisphere indicated by arrow). Phenotype ID17: linear cerebriform nevus before **(C)** and after **(D)** surgical excision. Phenotype ID18: linear epidermal nevus on the scalp and in the right temporal region **(E)**. For comparison, a congenital linear epidermal nevus in the right temporal region in a patient with Cowden syndrome is presented **(F)**.

Patient ID17, a 2-year-old boy (Figures 6C,D), was born with a hairless, well-defined linear plaque on the scalp, with no other abnormalities. Surgical excision of the skin lesion with subsequent plastic surgery of the right auricle was performed, and histology confirmed a cerebriform nevus. Genetic testing of the nevus revealed a pathogenic *FGFR2* variant, c.1144T>C (p.Cys382Arg), with a VAF of 37%. He was diagnosed with a rare subtype of cutis verticis gyrata, and at follow-up, no other pathologies were found.

Patient ID18, a 14-year-old girl (Figure 6E), was born with an EN on the right scalp. No other organ involvement was detected during follow-up. At 14 years of age, surgical excision of the nevus was performed, and genetic testing did not reveal any pathogenic/likely pathogenic variants in the genes studied.

### 3.4 Spectrum and frequency of pathogenic/likely pathogenic variants identified in the study

The summarized results of genetic testing for all patients and different tissue samples are presented in Supplementary Table S3. Pathogenic/likely pathogenic variants in the genes *HRAS* (n = 8), *KRAS* (n = 6), *BRAF* (n = 1), *PIK3CA* (n = 1), and *FGFR2* (n = 1) were present only in affected tissues (nevus and tumor) and were absent from peripheral blood DNA and unaffected skin. The detection limit for NGS was ≥2%. Variant allele frequencies ranged from 17% to 84% in tissue samples, whereas no variants were detected in peripheral blood samples or normal skin biopsies.

All candidate variants were validated by Sanger sequencing. However, we acknowledge that Sanger sequencing has a practical detection limit of approximately 15%–20% VAF; consequently, low-level mosaic variants with VAF below this range were not independently confirmed by Sanger and are reported based on NGS findings alone.

## 4 Discussion

The differential diagnosis of mosaic RASopathies remains a significant challenge for clinicians due to the rarity of these conditions and the similarity of their phenotypes. Typically, the first sign is characteristic skin lesions in the form of extensive epidermal nevi, which appear primarily in the craniofacial region. It is crucial for both parents and clinicians to understand the spectrum of possible clinical manifestations during the child’s development and discuss the individual patient’s prognosis. The primary factor believed to influence disease severity is the timing of the occurrence of postzygotic pathogenic/likely pathogenic variants during embryogenesis. This mainly determines the extent of tissue and organ involvement—whether ectodermal, mesodermal, or endodermal, or a combination thereof—and, consequently, the spectrum and expressivity of syndromic clinical manifestations (Groesser et al., 2012; Jaeger et al., 2025; Kinsler et al., 2020).

We also examined the variability in clinical manifestations and disease course in relation to the specific gene mutated, drawing on both our own data and the published literature. In our study, eight patients were diagnosed with SFMS and seven with PPK. In both groups, pathogenic variants in the *HRAS* or *KRAS* genes were predominant. We combined these two clinical groups and compared our results with those from previously published studies describing SFMS and PPK cases (Lim et al., 2014; Igawa et al., 2016; Kuroda et al., 2015; Wu et al., 2025; Morren et al., 2024; Chang et al., 2021; Davies et al., 2022; Vera-Duarte et al., 2025; Mirza et al., 2025; Del Pino et al., 2025; Ohishi et al., 2024; Hanna et al., 2025; Dionysiou et al., 2024; Querzoli et al., 2024; Tumova et al., 2024; Haller et al., 2024; Beyens et al., 2023; Miao et al., 2023; Yeager et al., 2024; D'Onofrio et al., 2023; Ono et al., 2023; Carli et al., 2022; Hafner and Groesser, 2013; Blanco et al., 2022; Huang et al., 2022; Green et al., 2021; Luo et al., 2021; Slack et al., 2021; De Los Santos-La Torre et al., 2020; Chang et al., 2021; Khadora and Mughal, 2021; Kapoor et al., 2021; Pepi et al., 2021; Sideris et al., 2021; Yan et al., 2021; Hayani et al., 2020; Pan et al., 2020; Friedrich et al., 2022; Chaves et al., 2020; Maia et al., 2020; Hoxha et al., 2020; Mestach et al., 2020; Gadea et al., 2020; Chacon-Camacho et al., 2019; Nagatsuma et al., 2019; Mitchel et al., 2019; Kubo and Yamada, 2019; Cranwell et al., 2019; Nemeth et al., 2018; Bessis et al., 2018; Park et al., 2018; Prieto-Barrios et al., 2018; Martin et al., 2018; Lihua et al., 2017; Om et al., 2017; Jennings et al., 2017; Whyte et al., 2017; Igawa et al., 2016; Hucthagowder et al., 2016; Ayala et al., 2016; Kuentz et al., 2016; Watanabe et al., 2016; Wang et al., 2015; Kim et al., 2015; Kuroda et al., 2015; Farschtschi et al., 2015; Lim et al., 2014; Li et al., 2014; Avitan-Hersh et al., 2014; Sun et al., 2013; Groesser et al., 2013; Rijntjes-Jacobs et al., 2010; Chantorn and Shwayder, 2011; Hafner et al., 2011; Bourdeaut et al., 2010; Wiedemeyer and Hartschuh, 2009; Zutt et al., 2003; Torrelo and Zambrano, 1998) to analyze the spectrum of gene variants, the variability of cutaneous and extracutaneous anomalies, and genotype–phenotype correlations (Supplementary Table S1).

In our study, the most common substitution in *HRAS* was Q61R (50%), followed by G13R (37%), whereas in the literature, the most frequent substitution was *HRAS* G13R (57%). The G12D substitution predominated among all *KRAS* pathogenic variants, both in our study (83%) and in cases from the literature (64%) (Figure 7A).

**FIGURE 7:**
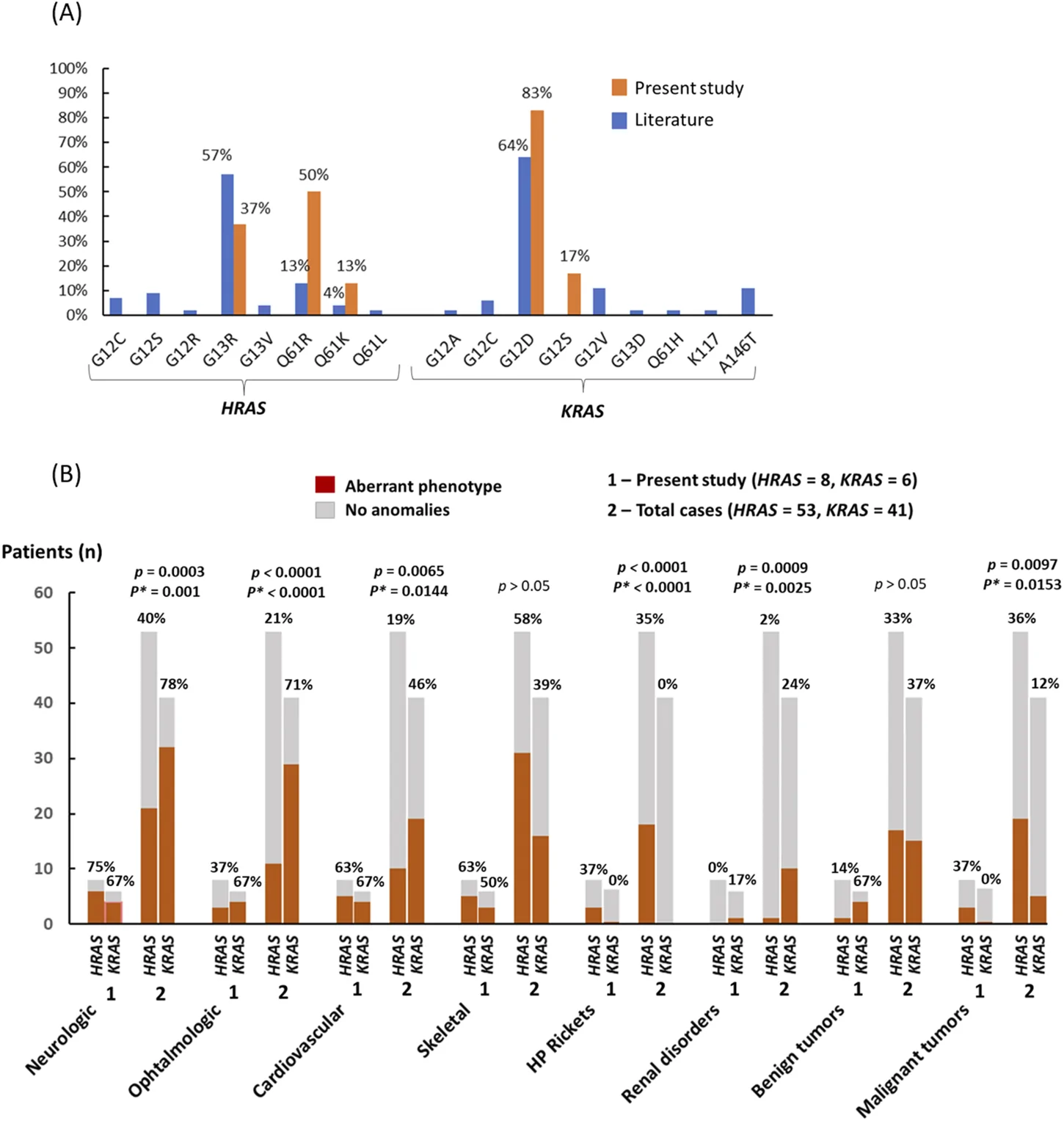
**(A)** Frequency of *HRAS* and *KRAS* genetic variants in the present study compared with the published literature data. **(B)** Frequency of different extracutaneous abnormalities in *HRAS*- and *KRAS*-mutant patients with mosaic RASopathies (*p* < 0.05 is considered significant; p* = FDR-adjusted *p*-value).

All our patients with *HRAS* pathogenic variants had craniofacial LNS, and four of them (50%) also had nevus spilus. Other cutaneous anomalies included EN, melanocytic nevi, and café-au-lait spots. According to the literature, LNS was present in 67% of the 45 *HRAS*-mutated cases, often in combination with nevus spilus (40%), EN, and KEN (Zelenova et al., 2025a; Morren et al., 2024; Zelenova EE. et al., 2025). All six of our patients with *KRAS* pathogenic variants also had congenital craniofacial LNS, and two of them had nevus spilus. Other skin lesions included café-au-lait spots and angiofibromas. In the 35 *KRAS*-mutated cases from the literature, LNS was noted in 68%, and only one patient also had nevus spilus, a finding indicative of PPK.

The analysis of extracutaneous manifestations revealed overlapping phenotypes between *HRAS*- and *KRAS*-mutated patients; however, the frequency of some extracutaneous anomalies differed between the two groups (Supplementary Table S2; Figure 7B). The most notable difference was the association of *KRAS* pathogenic/likely pathogenic variants with ophthalmic disorders (71% in *KRAS*-mutated vs. 21% in *HRAS*-mutated, OR = 9.2, 95% CI = 3.6–23.8, *p* < 0.0001, p* < 0.0001); coloboma, choristoma, and epibulbar/bulbar dermoid were detected only in patients with *KRAS* pathogenic/likely pathogenic variants, both in our study and in the literature (Supplementary Table S2). Neurological disorders (including epilepsy and various brain malformations) were also more common in *KRAS*-mutated than in *HRAS*-mutated patients (78% vs. 40%, OR = 5.4, 95% CI = 2.2–13.6, *p* = 0.0003, p* = 0.001), as were cardiovascular disorders and lymphatic malformations (46% vs. 19%, OR = 3.7, 95% CI = 1.5–9.3, *p* = 0.0065, p* = 0.0144; and 22% vs. 4%, OR = 7.2, 95% CI = 1.5–35.3, *p* = 0.009, p* = 0.015, respectively).

In our cases and in the literature, there is a strong association between *HRAS* pathogenic/likely pathogenic variants and HR, which was diagnosed exclusively in *HRAS*-mutated patients, occurring in 30%–40% of cases (p < 0.0001, p* <0.0001) (Lim et al., 2014; Wu et al., 2025; Mirza et al., 2025; Del Pino et al., 2025; Khadora and Mughal, 2021; Maia et al., 2020; Park et al., 2018; Hucthagowder et al., 2016). Other skeletal abnormalities (fibrous dysplasia, scoliosis, limb asymmetry, cystic bone lesions, *etc.*) were found in *HRAS*- and *KRAS*-mutated patients, and the difference was not statistically significant.

In the total sample, benign neoplasms occurred with equal frequency in both groups (33% in *HRAS*-mutated patients and 37% in *KRAS*-mutated patients), whereas malignancies were more frequent in the *HRAS*-mutated group (36% vs. 12%, OR = 4.0, *p* = 0.0097, p* = 0.0153). Among the malignancies, embryonal/alveolar RMS was more common in childhood (Davies et al., 2022; Mirza et al., 2025; Luo et al., 2021; Maia et al., 2020; Nemeth et al., 2018; Prieto-Barrios et al., 2018), while skin cancer (Hayani et al., 2020; Maia et al., 2020; Hoxha et al., 2020; Kubo and Yamada, 2019; Cranwell et al., 2019; Zutt et al., 2003), urothelial carcinoma (Gadea et al., 2020; Hafner et al., 2011), and astrocytoma (Tumova et al., 2024) have been described in adults. In our study, two *HRAS-*mutated patients (ID3 and ID4) developed RMS, and one patient (ID9) had ectomesenchymoma; all tumors occurred in the groin area before the age of 3 years, and the pathogenic variant had the highest VAF in the tumor tissue (84%, 76%, and 41%, respectively). A similar result when comparing allele frequencies in tumor and nevus tissues was observed in a previous study. Luo et al. (2021) explained this by a two-stage mechanism: a mutation in the *HRAS* gene may be the driving event behind the development of rhabdomyosarcoma, and the subsequent loss of heterozygosity may represent the second hit (Luo et al., 2021).

The rare case of malignant ectomesenchymoma in patient ID9 deserves special attention. A previous study described a patient with PPK combined with RMS and the same pathogenic variant in the *HRAS* gene (Prieto-Barrios et al., 2018). Given that malignant ectomesenchymoma tissue includes a mesenchymal component, which is most often represented by embryonic RMS, the association between this tumor type and the pathogenic *HRAS* variant may not be coincidental.

*KRAS* pathogenic/likely pathogenic variants have previously been described in Wilms’ tumor (Slack et al., 2021), embryonal RMS (Chang et al., 2021; Om et al., 2017; Bourdeaut et al., 2010), and yolk sac tumor (Mitchel et al., 2019), all occurring before the age of 1 year. In addition to nephroblastoma, renal anomalies (cysts, hamartomas, angiolipomas, *etc.*) were a very characteristic feature of *KRAS*-associated mosaicism (ID5) (Chang et al., 2021; Ohishi et al., 2024; Dionysiou et al., 2024; D'Onofrio et al., 2023; Bourdeaut et al., 2010), as was CPP (ID5 in our sample) (De Los Santos-La Torre et al., 2020; Martin et al., 2018; Kuentz et al., 2016). Hearing loss (Chang et al., 2021; Beyens et al., 2023; D'Onofrio et al., 2023; Hoxha et al., 2020; Chacon-Camacho et al., 2019; Nagatsuma et al., 2019; Watanabe et al., 2016; Wiedemeyer and Hartschuh, 2009) has been reported in both groups with equal frequency (Supplementary Table S2).

We have previously suggested that the expression of genetic information and the development of specific phenotypic patterns in mosaic syndromes may depend not only on the timing of the mutation but also on the expression levels of different genes of the RAS family in various organs and tissues during embryonic development (Zelenova et al., 2025a). In this study, we observed very similar genotype–phenotype correlations when using a larger sample. However, some comparison groups had small numbers of participants, so the results obtained can be considered exploratory and require further verification in replication studies. It should also be noted that the differences in the approaches and methods used across different studies in the literature may introduce uncertainty into the final estimates of associations. Further research in this area may help elucidate this question.

The differential diagnosis of ENS associated with postzygotic pathogenic/likely pathogenic variants in the RAS/MAPK pathway is challenging, and DNA testing plays a key role in establishing the diagnosis. Germline *BRAF* pathogenic/likely pathogenic variants can lead to CFC (Di Majo et al., 2025), while postzygotic variants have been described in PPK (Sorlin et al., 2021; Ayala et al., 2016; Watanabe et al., 2016). Patient ID15 had severe psychomotor delay, minor cardiac abnormalities, and facial dysmorphism, which are characteristic of CFC, as well as immunodeficiency of undetermined etiology. On the other hand, a diagnosis of PPK could also be considered based on the mosaic *BRAF* variant, congenital LNS, and nevus spilus. Patients with PROS may also have EN, in addition to asymmetric tissue overgrowth (Roth et al., 2018). In patient ID16 with EN, lower limb asymmetry, and hemimegalencephaly, SFMS was suspected, but the presence of a mosaic *PIK3CA* variant allowed the correct diagnosis of PROS to be made. Germline variants in the *FGFR2* gene may be associated with severe skeletal disorders (Zhuang et al., 2022), while mosaic variants can lead to phacomatosis resembling SFMS (Wang et al., 2025). The somatic pathogenic variant *FGFR2* c.1144T>C (VAF 23%–40%) was found in 5/8 patients with cerebriform nevus of the sebaceous glands (Theiler et al., 2021) and also in patient ID17 in our study. It is also important to exclude germline pathogenic/likely pathogenic variants in patients with mosaic RASopathies. Previously, we described a patient with a congenital facial epidermal nevus (Figure 6F) who was found to have a pathogenic *PTEN* variant first in the nevus, which was subsequently confirmed by blood DNA analysis (Zelenova et al., 2025c). Thus, the final diagnosis was type 2 segmental Cowden disease, which is associated with linear Cowden nevus, and lifelong surveillance was prescribed due to the high cancer risk.

In the nevus tissue of patient ID18, genetic analysis did not reveal any pathogenic/likely pathogenic variants in any of the genes associated with ENS. Furthermore, the patient had only skin manifestations, which were successfully corrected by surgery, and during further follow-up (more than 5 years), no symptoms characteristic of ENS were noted. Therefore, the case was considered an isolated skin malformation.

Based on this study, we propose the following algorithm for accurately determining somatic mosaicism in ENS. Given the varying VAFs in affected tissues, biopsies should be taken from at least four different areas of the nevus (and different types of nevi, if feasible), and genetic testing of peripheral blood should be performed to exclude germline pathogenic/likely pathogenic variants. This approach can help differentiate mosaic disorders from hereditary syndromes with incomplete phenotypic manifestations. It should also be borne in mind that Sanger sequencing has a limited ability to detect low-level allelic variants; therefore, next-generation sequencing is the method of choice for analyzing somatic mosaicism. A pathogenic/likely pathogenic variant in a specific gene can certainly influence the range of clinical manifestations; however, it is important to recognize that these correlations are probabilistic in nature. Therefore, all patients with characteristic skin lesions (epidermal nevi) and suspected mosaic forms of the disease should be referred for assessment by a multidisciplinary team of specialists (Morren et al., 2024).

This study has some limitations. Due to the rarity of these mosaic diseases, we had a limited number of cases for the statistical analysis. The comparison of small patient groups led to wide confidence intervals, indicating some inconsistency in the results. Another aspect is that extracutaneous anomalies can develop over a long period, while some of the patients in our study and in the literature cases were only 0–3 years of age. Therefore, certain syndromic manifestations may not yet have become fully apparent. It is also worth noting that in the cases reported in the literature, not all patients underwent a comprehensive examination of all organ systems, which could also have influenced the final statistical calculations. Further investigations involving more genetically confirmed cases will make it possible to identify clearer correlations and the molecular basis underlying the diverse phenotypic patterns.

## 5 Conclusion

Genetic testing plays a crucial role in accurately diagnosing mosaic forms of the disease. The spectrum and severity of clinical manifestations of mosaic RASopathies may depend not only on the timing of the postzygotic pathogenic/likely pathogenic variants but also on the specific gene involved. Based on this study, we propose a probabilistic genotype–phenotype correlation: patients with *KRAS*-mediated RASopathy are likely to have an increased risk of ophthalmological disorders (especially coloboma or choristoma), along with neurological, renal, and vascular abnormalities, while *HRAS*-mediated mosaic disorder is more likely to be associated with hypophosphatemic rickets and malignancies in both children and adults. However, this hypothesis needs to be confirmed in future studies involving larger and more thoroughly examined patient cohorts. Nevertheless, the specific mosaic genotype may, with some caution, be taken into consideration for prognosis, patient management, and the development of targeted therapies.

## Data Availability

The datasets presented in this study can be found in online repositories. The names of the repository/repositories and accession number(s) can be found in the article/Supplementary Material.
