# Supplementary material for: Mosaic RASopathies: comprehensive genetic and clinicopathological analysis for diagnosis and management
Source: Front Cell Dev Biol. 2026 Oct 15;14:1908228. doi: 10.3389/fcell.2026.1908228 (PMC13716791; doi:10.3389/fcell.2026.1908228)
Supplement: Supplementary file 1 [file Table2.docx]

**Table S2.** Extracutaneous disorders in *HRAS*-mutant and *KRAS*-mutant patients with mosaic RASopathies. OR – odds ratio, CI – confident interval, the difference is statistically significant at *p* < 0.05. *p** - FDR-adjusted p-value (FDR -False Discovery Rate). ORs were calculated for the comparison of *KRAS* versus *HRAS*, such that OR > 1 indicates a higher prevalence in *KRAS*-mutant patients.

| **Extracutaneous anomalies** | ***HRAS* mutation** | | | ***KRAS* mutation** | | | **OR** | **95% CI** | ***p*-value** | ***p**-value (FDR-adjusted)** |
| --- | --- | --- | --- | --- | --- | --- | --- | --- | --- | --- |
|  | **Present study**  **(n=8)** | **Literature**  **(n=45)** | **Total**  **(n=53)** | **Present studу**  **(n=6)** | **Literature**  **(n=35)** | **Total**  **(n=41)** |  |  |  |  |
| **Neurologic** | | | | | | | | | | |
| **Present** | **6 (75%)** | **15 (33%)** | **21 (40%)** | **4 (67%)** | **28 (80%)** | **32 (78%)** | **5.4** | **2.2–13.6** | **0.0003** | **0.001** |
| **Not found** | **2 (25%)** | **30 (67%)** | **32 (60%)** | **2 (33%)** | **7 (20%)** | **9 (22%)** |  |  |  |  |
| Epilepsy, seizures | 3 (37%) | 7 (16%) | 10 (19%) | 4 (67%) | 14 (40%) | 18 (44%) |  |  |  |  |
| Intellectual disability | 3 (37%) | 7 (16%) | 10 (19%) | 4 (67%) | 10 (29%) | 14 (34%) |  |  |  |  |
| Brain anomalies | 1 (12%) | 7 (16%) | 8 (15%) | 3 (50%) | 20 (57%) | 23 (56%) |  |  |  |  |
| **Ophtalmologic** | | | | | | | | | | |
| **Present** | **3 (37%)** | **8 (18%)** | **11 (21%)** | **4 (67%)** | **25 (71%)** | **29 (71%)** | **9.2** | **3.6–23.8** | **0.0000** | **0.0000** |
| **Not found** | **5 (63%)** | **37 (82%)** | **42 (79%)** | **2 (33%)** | **10 (29%)** | **12 (29%)** |  |  |  |  |
| Coloboma/choristoma/dermoids | - | - | - | 4 (67%) | 16 (46%) | 20 (49%) |  |  |  |  |
| Other anomalies | 2 (25%) | 8 (18%) | 10 (19%) | 3 (50%) | 18 (51%) | 21 (51%) |  |  |  |  |
| **Cardiovascular** | | | | | | | | | | |
| **Present** | **5 (63%)** | **5 (11%)** | **10 (19%)** | **4 (67%)** | **15 (43%)** | **19 (46%)** | **3.7** | **1.5–9.3** | **0.0065** | **0.0144** |
| **Not found** | **3 (37%)** | **40 (89%)** | **43 (81%)** | **2 (33%)** | **20 (57%)** | **22 (54%)** |  |  |  |  |
| **Lymphatic malformation/lymphedema** | | | | | | | | | | |
| **Present** | **-** | **2 (4%)** | **2 (4%)** | **-** | **9 (26%)** | **9 (22%)** | **7.2** | **1.5–35.3** | **0.009** | **0.0153** |
| **Not found** | **8 (100%)** | **43 (96%)** | **51 (96%)** | **6 (100%)** | **26 (74%)** | **32 (78%)** |  |  |  |  |
| **Skeletal and muscular** | | | | | | | | | | |
| **Present** | **5 (63%)** | **26 (58%)** | **31 (58%)** | **3 (50%)** | **13 (37%)** | **16 (39%)** | 0.5 | 0.2–1.0 | 0.0956 | 0.1314 |
| **Not found** | **3 (37%)** | **19 (42%)** | **22 (42%)** | **3 (50%)** | **22 (63%)** | **25 (61%)** |  |  |  |  |
| Rickets and HP | 3 (37%) | 15 (33%) | 18 (35%) | - | - | - | **0*** | **0.0–0.4** | **0.0000** | **0.0000** |
| Other anomalies | 4 (50%) | 19 (42%) | 22 (42%) | 3 (50%) | 13 (37%) | 16 (39% ) |  |  |  |  |
| **Reproductive system disorders** | | | | | | | | | | |
| **Present** | **2 (25%)** | **6 (13%)** | **8 (15%)** | **1 (17%)** | **2 (6%)** | **3 (7%)** | 0.4 | 0.1–1.8 | 0.3378 | 0.4129 |
| **Not found** | **6 (75%)** | **39 (87%)** | **45 (85%)** | **5 (83%)** | **33 (94%)** | **38 (93%)** |  |  |  |  |
| Precocious puberty | 1 (12% ) | 4 (9%) | 5 (9% ) | 1 (17%) | - | 1 (2%) |  |  |  |  |
| **Renal disorders (excluding tumors)** | | | | | | | | | | |
| **Present** | **-** | **1 (2%)** | **1 (2%)** | **1 (17%)** | **9 (26%)** | **10 (24%)** | **16.8** | **2.0–137.4** | **0.0009** | **0.0025** |
| **Not found** | **8 (100%)** | **44 (98%)** | **52 (98%)** | **5 (83%)** | **26 (74%)** | **31 (76%)** |  |  |  |  |
| Cyst of kidney | - | - | - | 1 (17%) | 6 (17%) | 7 (17%) |  |  |  |  |
| Other disorders | - | 1 (2%) | 1 (2%) | - | 3 (8%) | 3 (7%) |  |  |  |  |
| **Benign tumors** | | | | | | | | | | |
| **Present** | **1 (14%)** | **16 (36%)** | **17 (33%)** | **4 (67%)** | **11 (31%)** | **15 (37%)** | 1.2 | 0.5–2.9 | 0.6669 | 0.6950 |
| **Not found** | **7 (86%)** | **29 (64%)** | **36 (67%)** | **2 (33%)** | **24 (69%)** | **26 (63%)** |  |  |  |  |
| **Malignant tumors** | | | | | | | | | | |
| **Present** | **3 (37%)** | **16 (36%)** | **19 (36%)** | **-** | **5 (14%)** | **5 (12%)** | **0.25** | **0.1–0.7** | **0.0097** | **0.0153** |
| **Not found** | **5 (63%)** | **29 (64%)** | **34 (64%)** | **6 (100%)** | **30 (86%)** | **36 (88%)** |  |  |  |  |
| RMS | 2 (25%) | 4 (9%) | 6 (11%) | - | 3 (9%) | 3 (7%) |  |  |  |  |
| BCC, SCC | - | 7 (16%) | 7 (13%) | - |  | - |  |  |  |  |
| Urothelial carcinoma | - | 2 (4%) | 2 (4%) | - | - | - |  |  |  |  |
| Wilm’s tumor | - | - | - | - | 1 (3%) | 1 (2%) |  |  |  |  |
| Other tumors | 1 (12%) | 2 (4%) | 4 (8%) | - | 1 (3%) | 1 (2%) |  |  |  |  |
| **Hearing loss** | | | | | | | | | | |
| **Present** | **-** | **3 (7%)** | **3 (6%)** | **-** | **4 (11%)** | **4 (10%)** | 1.8 | 0.4–8.5 | 0.6950 | 0.6950 |
| **Not found** | **8 (100%)** | **42 (93%)** | **50 (94%)** | **6 (100%)** | **31 (89%)** | **37 (90%)** |  |  |  |  |

** - OR calculated with Haldane correction (+0.5) due to zero cells*
