# Supplementary material for: Mosaic RASopathies: comprehensive genetic and clinicopathological analysis for diagnosis and management
Source: Front Cell Dev Biol. 2026 Oct 15;14:1908228. doi: 10.3389/fcell.2026.1908228 (PMC13716791; doi:10.3389/fcell.2026.1908228)
Supplement: Supplementary file 2 [file Table3.docx]

| **#** | **Gene** | **Transcript** | **HGVS, protein** | **ClinVar/ACMG** | **Sample** | **Total R/**  **Alt R** | **VAF, %** |
| --- | --- | --- | --- | --- | --- | --- | --- |
| ID1 | *HRAS* | NM_005343.4 | c.182A>G, Q61R | LP/P | LNS | 304/188 | 62% |
|  |  |  |  |  | Blood | 223/0 | 0% |
| ID2 | *HRAS* | NM_005343.4 | c.182A>G, Q61R | LP/P | LNS (1) | 435/121 | 28% |
|  |  |  |  |  | LNS (2) | 451/163 | 36% |
|  |  |  |  |  | Blood | 200/0 | 0% |
| ID3 | *HRAS* | NM_005343.4 | c.37G>C, G13R | P/P | LNS | 460/101 | 22% |
|  |  |  |  |  | RMS | 378/317 | 84% |
|  |  |  |  |  | Blood | 345/0 | 0% |
| ID4 | *HRAS* | NM_005343.4 | c.181C>A, Q61K | LP/P | LNS (1) | 457/82 | 18% |
|  |  |  |  |  | LNS (2) | 565/209 | 37% |
|  |  |  |  |  | RMS | 234/177 | 76% |
|  |  |  |  |  | Blood | 256/0 | 0% |
| ID5 | *KRAS* | NM_004985.5 | c.35G>A, G12D | P/P | LNS | 435/152 | 35% |
|  |  |  |  |  | Blood | 234/0 | 0% |
| ID6 | *KRAS* | NM_004985.5 | c.35G>A, G12D | P/P | LNS (1) | 251/57 | 23% |
|  |  |  |  |  | LNS (2) | 252/70 | 28% |
|  |  |  |  |  | Blood | 339/0 | 0% |
| ID7 | *KRAS* | NM_004985.5 | c.35G>A, G12D | P/P | EN | 435/143 | 33% |
|  |  |  |  |  | Blood | 367/0 | 0% |
| ID8 | *KRAS* | NM_004985.5 | c.35G>A, G12D | P/P | EN | 308/92 | 30% |
|  |  |  |  |  | Skin (N) | 320/0 | 0% |
|  |  |  |  |  | Blood | 342/0 | 0% |
| ID9 | *HRAS* | NM_005343.4 | c.37G>C, G13R | P/P | LNS | 548/197 | 36% |
|  |  |  |  |  | EMS | 564/231 | 41% |
|  |  |  |  |  | Blood | 453/0 | 0% |
| ID10 | *HRAS* | NM_005343.4 | c.37G>C, G13R | P/P | LNS | 534/197 | 37% |
|  |  |  |  |  | Blood | 456/0 | 0% |
| ID11 | *HRAS* | NM_005343.4 | c.182A>G, Q61R | LP/P | LNS | 234/ | 33% |
|  |  |  |  |  | Blood | 343/0 | 0% |
| ID12 | *HRAS* | NM_005343.4 | c.182A>G, Q61R | LP/P | LNS | 543/108 | 20% |
|  |  |  |  |  | NS | 445/111 | 25% |
|  |  |  |  |  | Blood | 324/0 | 0% |
| ID13 | *KRAS* | NM_004985.5 | c.35G>A,  G12D | P/P | LNS | 234/74 | 32% |
|  |  |  |  |  | Blood | 434/0 | 0% |
| ID14 | *KRAS* | NM_004985.5 | c.34G>A,  G12S | P/P | LNS | 348/104 | 30% |
|  |  |  |  |  | Blood | 245/0 | 0% |
| ID15 | *BRAF* | NM_004333.6 | c.1406G>C,  G469A | P/LP | LNS | 324/84 | 26% |
|  |  |  |  |  | Blood | 251/0 | 0% |
| ID16 | *PIK3CA* | NM_006218.4 | c.1633G>A,  Q545K | P/P | EN | 379/109 | 29% |
|  |  |  |  |  | Blood | 324/0 | 0% |
| ID17 | *FGFR2* | NM_000141.5 | c.1144T>C, C382R | LP/P | Nevus | 215/79 | 37% |
|  |  |  |  |  | Blood | 203/0 | 0% |
| ID18 | None | None | None | None | Nevus | 514/0 | 0% |
|  |  |  |  |  | Blood | 442/0 | 0% |

**Table S3.** Pathogenic/likely pathogenic variants identified in the study. LP – likely pathogenic, P – pathogenic, LNS – linear nevus sebaceous, EN – epidermal nevus, NS – nevus spilus, Total R – total reads, Alt R – alternate reads, VAF – variant allele frequency, RMS – rhabdomyosarcoma, EMS – ectomesenchymoma N - normal.
